# A cross-sectional assessment of expanding basic healthcare services to rural and underserved communities through proprietary patent medicine vendors in Northern Nigeria

**DOI:** 10.1371/journal.pgph.0003671

**Published:** 2024-09-20

**Authors:** John Oluwasegun Ibitoye, Olugbenga Asaolu, Adebayo Amao, Oluwagbemiga Obembe, Mukhtar A. Ijaya, Christopher Obanubi, Adeniyi Adeniran, Mustapha Bello, Olutayo Asaolu, Gbadegesin Alawode, Chiamaka Uwalaka, Olubunmi Ojelade, Chisom Emeka, Bayo Onimode, Olubayode Asaolu, Titus Ojewumi, Nannim Nalda, Olubusola Oyeyemi, Abdulmalik Abubakar, Chukwuka Nwadike, Adaeze Ugwu

**Affiliations:** 1 Department of Public Health, Texila American University, Guyana, Nicaragua; 2 Department of Public Health, Babcock University, Ilishan-Remo, Nigeria; 3 College of Medicine, Lagos State University, Lagos, Nigeria; 4 Data-Lead Africa, Abuja, Nigeria; 5 School of Public Health, University of the Western Cape, Cape Town, South Africa; 6 Nuffield Center for International Health and Development, University of Leeds, Leeds, United Kingdom; 7 Department of Business Administration, Babcock University, Ilishan-Remo, Nigeria; 8 Department of Programs, Association for Reproductive and Family Health, Lagos, Nigeria; 9 Institute for Development Studies, University of Nigeria, Nsukka, Nigeria; 10 Department of Microbiology, University of Ilorin, Ilorin, Nigeria; 11 Department of Demography and Social Statistics, Obafemi Awolowo University, Ile-Ife, Nigeria; 12 School of Health and Social care, Staffordshire University, Stoke-on-Trent, United Kingdom; University of Global Health Equity, RWANDA

## Abstract

The poor health indices in Nigeria are widely reported to be fueled by an acute shortage of skilled medical personnel. Opinions are converging that Proprietary and Patent Medicine Vendors (PPMVs) can bridge this human resource for health gaps. This study therefore aimed to assess the acceptability, appropriateness, and feasibility of providing expanded basic health services among the skilled health workers operating PPMVs in underserved communities in Northern Nigeria states. This is a cross-sectional study of all the 220 PPMVs operated by skilled health workers in underserved communities of six randomly selected Local Government Areas in Jigawa state) and Kaduna State from July to October 2022). Statistical significance was determined at P<0.05. Of the 220 respondents surveyed, 77% are males; the median age was 33 years (IQR = 9). More than half (52.3%) were community health extension workers, and 16.4% are nurses/midwives. The median scores (with IQR) for Acceptability, Appropriateness, and Feasibility were 18 (16), 17 (15), and 17 (15), respectively. We reported that PPMVs of tribes other than Hausa, Fulani or Kanuri; operating their shops in the evening or morning or possessed positive behavioral control expressed lower acceptability (P< 0.05). Operating in Kaduna State and perceived negative behavioral control were significantly associated with lower appropriateness measure(P<0.01). PPMVs operating below 24 hours had higher appropriateness (P<0.01). PPMVs operating below 24 hours and perceived negative behavioral control had lower feasibility scores. Our Study findings suggests that there is significant potential for medically trained PPMVs operating in underserved communities in Northern Nigeria to contribute to bridging the gap in access to basic health services in hard-to-reach areas.

## Introduction

The persistent and harmonious efforts of Ministries, Departments, Agencies of Government at Federal and State levels and other development partners and stakeholders have led to gradual but slow improvements in healthcare access and outcomes in Nigeria. From 1990 to 2018, the under-five mortality rate in Nigeria declined from 193 to 132 child deaths per 1,000 live births [1] However, this slow progress has been undermined by disparities in gender, rural-urban distribution, and regional inequalities in healthcare resources, including facilities and workforce [2], coverage of services [1] and, consequently, health outcomes [3].

Regional disparities in healthcare access are a major drawback for Nigeria’s healthcare system. For instance, in the Northwest geopolitical zone of Nigeria, only 53.9% of antenatal care (ANC) visits were attended by a skilled provider, compared to the national average of 67% [1]. Similarly, only 18.2% of births were attended by a skilled provider, significantly lower than the national average of 43.3% [1]. Additionally, only 19.9% of children aged 12–23 years were fully immunized, below the 31% national average. Rural-urban variations are also stark, as exemplified by the proportion of pregnant women who did not attend any hospital for antenatal care in their last pregnancy—10.1% in urban areas compared to 33.8% in rural settings [1]. Gender disparity in health services remains a critical issue, particularly in the realm of family planning and maternal care. Oduenyi and colleagues [4], in a study conducted in two Nigerian states, reported significant biases and harmful practices within the healthcare system. For instance, 60% of healthcare providers believe that women cannot choose a family planning method without their male partner’s involvement. Additionally, 23.2% of providers are against unmarried clients using family planning methods. Furthermore, harmful practices were reported in 59.6% of deliveries, while disrespectful or abusive behaviors were observed in 34.0% of these cases.

Approximately half of Nigeria’s 216.8 million population resides in rural areas [5] these regions suffer from limited access to healthcare facilities and providers [6] Rural healthcare facilities often lack basic infrastructure such as electricity, clean water, and medical equipment, severely compromising the quality and availability of services [7,8] The maldistribution of healthcare professionals further aggravates access and quality healthcare service challenges, with a concentration of professionals in urban centers leaving rural communities underserved [9,10].

Economic barriers also prevent rural residents from accessing healthcare services. Studies have indicated that poverty and high out-of-pocket expenses significantly hinder healthcare utilization [11,12] perpetuating health inequalities and cycles of poverty. Moreover, cultural beliefs, norms, and sociodemographic factors such as gender disparities and literacy levels influence healthcare-seeking behaviors and access in rural Nigeria [13–15].

Policy deficiencies and inadequate governance structures also contribute to these systemic challenges in rural healthcare systems [16,17]. However, community-based healthcare interventions, such as those involving community health workers, mobile clinics, and health education programs, have shown promise in improving access to basic quality healthcare services and reducing disparities [18–20].

A proprietary and patent medicine vendor (PPMV) is defined as “a person without formal training in pharmacy who sells orthodox pharmaceutical products on a retail basis for profit” [21]. Proprietary and Patent Medicine vendors (PPMVs) play a crucial role in healthcare delivery, especially in rural areas. There are over 20,000 registered PPMVs and Community Pharmacy outlets in Nigeria, with approximately 40% operated by skilled health workers. These vendors often serve as the first point of contact for healthcare in underserved communities [22–25].

Despite concerns regarding the quality of care, the relative abundance, and medical skills of PPMVs provide a vital healthcare resource in rural areas. In light of this, the Government of Nigeria, through the Pharmacy Council of Nigeria (PCN), initiated a pilot three-tier accreditation system for PPMVs in selected states to expand access to basic healthcare services- such as injectable methods of family planning, diagnosis and treatment malaria, diarrhoea and other common childhood illnesses- which they are typically not licensed to provide within Patent Medicine Stores. This study aims to assess the acceptability, appropriateness, and feasibility of providing expanded basic healthcare services among medically trained PPMVs operating in rural underserved communities in Kaduna and Jigawa states, Nigeria. Our literature review did not find any documented findings on the perception of PPMVs in these underserved rural communities regarding the provision of such services, underscoring the importance of this research.

## Methods

### Ethics statement

The study was approved by the Ethics Research committees of the Kaduna and Jigawa state’s Ministries of Health with reference numbers MOH/ADM/744/VOL.1/1134 and MOH/SEC/I.S/657/V1 respectively. Participants were interviewed after completing and signing the informed consent form for the study.

### Study design

This was a descriptive cross-sectional study of PPMVs operated by either a community health worker or nurse/midwife in medically underserved communities in the Kaduna and Jigawa state of North-western region in Nigeria.

### Study setting

North-west Nigeria is one of the six geopolitical zones of Nigeria comprising 7 states (including Jigawa, Kaduna, Kano, Katsina, Kebbi, Sokoto, and Zamfara states). The region is native to Hausa and Fulani tribes. North-West states account for about 15% of Nigeria’s population [5]. The region has a high poverty rate, a low literacy rate, a high concentration of rural communities, and a low population density [26].

Kaduna State has a projected population of 8.8 million people in 2017 and 23.7% of the population are women of reproductive age (15–49 years) 19.2% are children under 5 years of age, 46.1% are children under the age of 15 years and 80.3% are below 35 years of age. With about 3.1% of Nigeria’s population, Jigawa State ranked 8th among the most populous states in the federation. Recent projections by the National Population Commission put the State’s population at about 5,828,163 in 2016, based on the 2006 Census. In terms of age distribution, about 47.2% are below the age of 15 years, 47.7% are between 15 and 59 years, and 5% are 60 years and above. That means, approximately 52% of the population are within the age bracket normally classified as “economically inactive.” There are 1,642 Medically trained PPMVs (650 in Jigawa and 992 in Kaduna) spread across both rural and urban communities in both states.

### Study population

The study population comprised all the PPMVs operated by Community Health Extension Workers (CHEW), Junior Community extension worker (JCHEW), Nurses, or Midwife in selected 9 LGAs of Kaduna and Jigawa States of Nigeria.

### Sampling strategy

The study employed a multistage sampling approach. Two states (Kaduna and Jigawa) were randomly selected from the 7 states in the North-western geopolitical zones(regions) through a simple balloting method. Based on the list of LGAs (9 in Kaduna, 15 in Jigawa) with hard-to-reach communities obtained from the State Ministries of health, 9 LGAs with the highest concentration of rural, hard to reach communities (top 30% in each state) were then purposively selected from the two states. This resulted in the selection of 6 LGAs in Jigawa (Birniwa, Gwaram, Kiri-Kasamma, Kiyawa, and Jahun) and 3 LGAs (Igabi, Kachia and Kubau) in Kaduna.

### Sample size

This study included all the 220 (115 in Kaduna, 105 in Jigawa) trained PPMVs in the 9 selected LGAs (3 in Kaduna, 6 in Jigawa).

### Measures

We used measures from 3 validated scales developed by Weiner et al., 2017 in this study. They include: (1) Acceptability of Intervention Measure (AIM) Scale; (2) Intervention Appropriateness Measure (IAM) Scale; Feasibility of Intervention Measure (FIM) Scale. The scales had 4 items each and were Likert-like with 5 ranking levels from strongly disagree (rated as “1”) to Strongly agree (rated as “5”), producing a maximum score of 20 and minimum of 4 for each measure. The scales were used in English language (their original language of development). The three scales have demonstrated acceptable psychometric properties in three previous studies [27] and applied in similar settings in Nigeria [28,29] and Ghana [30] Prior to data collection for this study, we pilot-tested the scales with 30 medically trained patent medicine vendors in Gwagwalada Area Council in Abuja (a setting similar to the study location). To assess the inter-rater reliability of our measurement scales, we employed the Intraclass Correlation Coefficient (ICC), a widely used measure for quantifying the degree of consistency among measurements made by different observers [31]. We found high ICC values for the three scales- AIM (0.978), IAM (0.967) and FIM (0.964). These results suggest that our measurement scales demonstrate robust inter-rater reliability, indicating that they can be used consistently across different raters. The scales also showed high internal consistency, recording high Cronbach Alpha values when assessed on the whole dataset for this study (AIM (0.86), IAM (0.93) and FIM (0.89)).

### Data collection instrument and process

A semi-structured 57-item instrument was digitized on KOBO toolbox (an open-source data collection platform) and administered by the trained Research Assistants on the medically trained PPMVs in Jigawa state (from July 2, 2020 to July 20, 2022) and Kaduna State (from July 15, 2022 to October 2, 2022). The instrument was used to collect information on the characteristics of the PPMV (including demographics of the PPMV, educational and professional qualification; years of practice); shop characteristics (location of shop, ‘age’ of shop, operating hours, types of services, clientele); Perceived behavioral control, Subjective Norm; acceptability of, and intention to adopt provision of expanded BHS (using the Intervention Appropriateness Measure, Acceptability of Intervention Measure and Feasibility of intervention Measure) scales. Subjective norm was assessed as a positive response to the question “*Most people would not have a problem with me providing expanded services*” and Perceived behavioral control was similarly evaluated as agreement with the statement “*I believe the decision to provide expanded basic health services is up to me and I don’t need confirmation from anyone*”.

Before finalization, the draft instrument was validated by sharing with five experts in the subject matter including Executives of the National Association of Patent and Proprietary Medicine Dealers (2); Public health officials with a history of working with the PPMVs (2) and one academic researcher who have previously researched the PPMVs.

### Data collection process & quality assurance

Eight Research Assistants (RAs) with prior experience in interviewing the PPMVs were engaged for data collection in each of the two states. Each of the RAs has at least a tertiary level education, were proficient in either Hausa or Fulfulde language RAs completed a three-day training, which orientated them on the data collection activities. The survey data was collected by 4 data collection teams, each including 2 pairs of data collectors and one trained supervisor. Supervisors clarified questions about the data collection tools and supported their administration throughout the data collection process. Given that data were collected with tablets on electronic forms, the supervisor was tasked to ensure that the data are regularly saved and uploaded (synched) to the encrypted cloud-based server.

### Data analysis

Data were cleaned and analyzed using the Stata version 18 (StataCorp, 2019; https://www.stata.com/), The descriptive statistics used included frequency counts, means, median, interquartile range, and graphical presentation. *Shapiro*–*Wilk test was used to ascertain* the non-normality of the distribution of the outcome variables—AIM, IAM and FIM scores (W = 0.72, p<0.001), See S1 Data for reference. We computed median scores and used Mann-Whitney-U and Kruskal Wallis tests to evaluate statistical association between the outcome measures and independent variables (sociodemographic, shop characteristics, profile of PPMV) with two or more categories respectively. The variables statistically associated with the outcomes were then included in Kernel regression models to identify the predictors for the respective outcome variables.

Kernel regression, a non-parametric method, was employed to explore potentially non-linear relationships between independent and dependent variables. Unlike linear models that provide global coefficients, kernel regression offers local estimates of these relationships [32]. Our analysis used the Nadaraya-Watson estimator with a Gaussian kernel function. For each predictor variable, we report:

(a) Local Estimates: These are weighted averages of nearby observations, with weights determined by the kernel function. They represent the estimated change in the outcome variable for a small change in the predictor at specific points in the data range. No transformations or approximations were applied to interpret these local estimates as global effects.

(b) Bootstrap Confidence Intervals: To assess the reliability of our local estimates, we performed 100 bootstrap resamples. The 95% confidence intervals derived from this process are reported alongside the local estimates (see Tables 3 to 5).

The computed local estimates provide insights into the relationships between variables at specific points in the data range. Statistical significance was determined at P-values less than 0.05.

## Results

### Profile of medically trained proprietary and patent medicine vendors (PMVs) surveyed

Of the 220 medically trained PPMVs interviewed (115 in Kaduna and 105 in Jigawa), 77.7% were male, the median age was 33 years(IQR = 9). Most, 52.3% of the respondents were community health extension workers by training (Table 1). Also, 71.4% were Hausa by tribe and 91.8% were married. In terms of the time of operation, 42.7% operate their outlets only in the daytime (8 am to 6 pm) and 37.3% operate 24 hours. Most of the PPMVs (61.8%) were still engaged within the health sector as at the time of data collection. Almost half (47.7%) of the respondents worked in government hospitals and 31(14.1%) were engaged in private facilities. About half (48.7%) of the outlets operated by medically trained persons have operated for more than five years. Only a few (3.6%) do not reside in the same community where their Patent medicine outlets are situated.

**Table 1:** Profile of medically trained PPMVs.

Characteristics	Kaduna State		Jigawa State		Total	
	Number	%	Number	%	Number	%
**Sex**						
Male	66	57.4	105	100.0	171	77.7
Female	49	42.6	0	0.0	49	22.3
**Age (Median, range)**	32 (IQR = 7) years		34 (IQR = 9) years		33 (IQR = 9) years	
22–34 years	62	53.9	44	41.9	106	48.2
35–44 years	44	38.3	47	44.7	91	41.4
45–65 years	9	7.8	14	13.3	23	10.4
**Professional Qualification**						
JCHEW*	38	33.0	17	16.2	55	25.0
CHEW**	45	39.1	70	66.7	115	52.3
CHO***	7	6.1	7	6.7	14	6.4
Nurse/Midwives	25	21.7	11	10.5	36	16.4
**Tribe**						
Hausa	77	66.9	80	76.2	157	71.4
Fulani/Kanuri	8	7.0	25	23.8	33	15.0
Others	30	26.1	0	0.0	30	13.6
**Marital Status**						
Married (Currently married)	102	88.7	100	95.2	202	91.8
Single (Never in a union)	13	11.3	5	4.8	18	8.2
**Operating Hours**						
Day time (8AM to 6PM)	39	33.9	55	52.4	94	42.7
Evening only (6PM upwards)	26	22.6	9	8.6	35	15.9
Morning only (8AM to 2 PM)	2	1.7	7	6.7	9	4.1
24 hours (Day and Night)	48	41.7	34	32.4	82	37.3
**Engaged in Other Business**						
None	34	29.6	8	7.6	42	19.1
Government Hospital	16	13.9	89	84.7	105	47.7
Private Hospital	29	25.2	2	1.9	31	14.1
Other Government work	3	2.6	4	3.8	7	3.18
Other jobs	33	28.7	2	1.9	35	15.9
**Age of Shop**						
Less than 1 year	14	12.2	0	0.0	14	6.4
One to three years	46	40.0	18	17.1	64	29.1
Four to Five years	23	20.0	12	11.4	35	15.9
Six to Seven years	10	8.7	26	24.8	36	16.4
Eight to Ten years	11	9.5	23	21.9	34	15.5
More than 10 years	11	9.5	26	24.8	37	16.8
**Resident in the Community**						
Yes	108	93.9	104	99.1	212	96.4
No	7	6.1	1	0.9	8	3.6
**Total Respondents**	**115**	**52.3**	**105**	**47.7**	**220**	**100.0**

*Junior Community Health Extension Workers

**Community Health Extension Workers

***Community Health Officers.

### Acceptability, appropriateness and feasibility of provision of expanded basic health services

**Fig 1:**
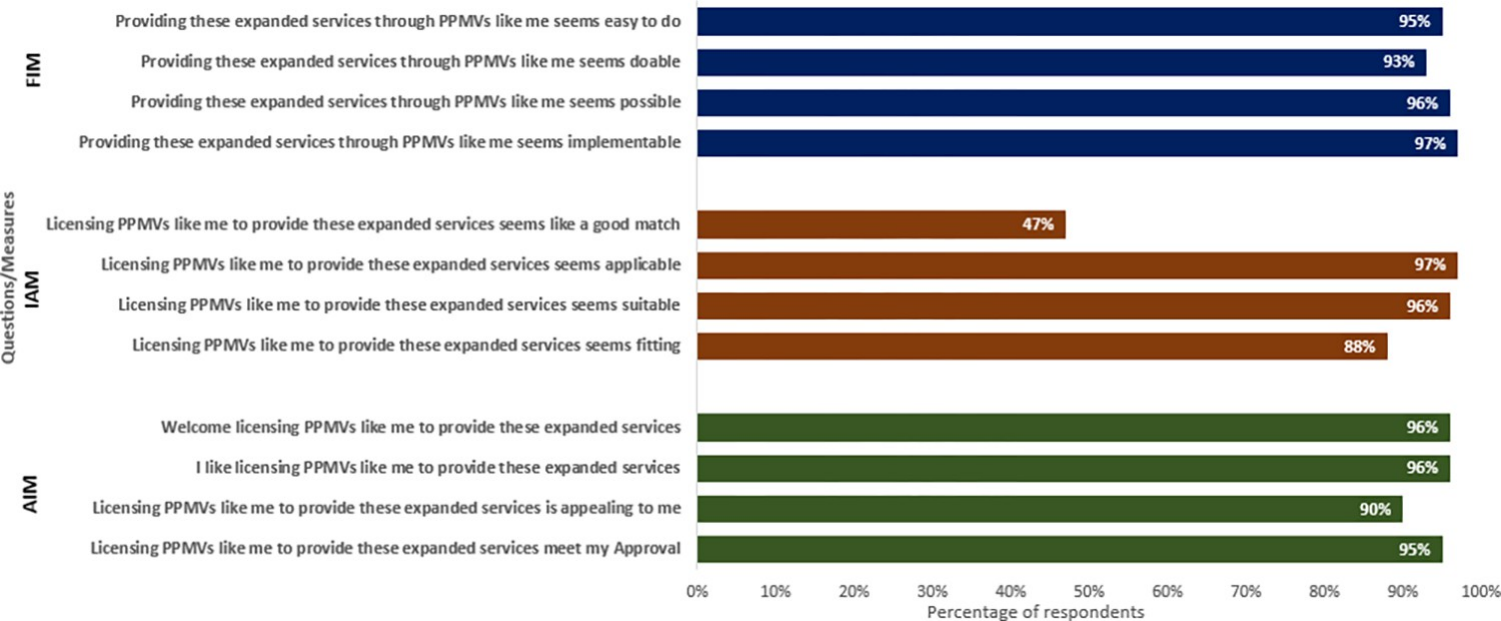
Responses to questions on acceptability, appropriateness and feasibility of providing expanded basic health services AIM: Acceptability of intervention measure; IAM: Intervention Appropriateness Measure: FIM: Feasibility of Intervention Measure.

Using the implementation measures (IAM, AIM, FIM), PPMVs shared their readiness to provide expanded basic health care services under an accreditation system. The median scores (and range) are as follows: AIM (18, IQR = 1620), IAM (17, IQR = 15), and FIM (17, IQR = 15). Fig 1 reports the proportion of respondents who agreed/completely agreed with survey questions about the acceptability, appropriateness, and feasibility of providing expanded basic health services. More respondents considered the provision of expanded BHS acceptable (93–97%) and Feasible (90–96%) than appropriate (47–97%). Specifically, only 47% feels that providing expanded basic health services seems like a good match.

Fig 1 (see attached additional files) shows the proportion of respondents who agreed/completely agreed with survey questions about acceptability, appropriateness, and feasibility of providing expanded basic health services. AIM, acceptability of intervention measure, IAM, intervention appropriateness measure; FIM, feasibility of intervention measure.

### Association between implementation measures and PPMVs and shop characteristics

The statistical association between implementation measures and PPMV characteristics are shown in Table 2. There was a statistically significant association between the acceptability of intervention measure score and the PPMV’s state of residence, tribe, operating hours, as well as perceived behavioral control (P <0.05). The same set of variables (PPMV’s state of residence, tribe, operating hours, as well as perceived behavioral control) were, in addition to sex of the PPMV, statistically associated with Intervention Appropriateness Measure (IAM) score (P< 0.05). Results also showed that Tribe, Operating hours and perceived behavioral control were statistically associated with Feasibility of Intervention Measure (FIM) score(P<0.05).

**Table 2:** Association between PPMV characteristics and acceptability, feasibility and appropriateness of providing expanded BHS.

Variables	Freq(n)	Acceptability			Appropriateness			Feasibility		
		Median	Statistic	P-value	Median	Statistic	P-value	Median	Statistic	P-value
**State of Residence**										
Jigawa	105	16	2.706	0.007*	15	3.69	0.001*	16	1.553	0.121
Kaduna	115	18			17			17		
**Sex**										
Female	49	18	1.706	0.089	17	1.98	0.048*	17	0.453	0.651
Male	171	**18**			17			17		
**Professional Qualification**										
JCHEW	55	18	1.802	0.615	17	3.275	0.351	17	0.132	0.988
CHEW	115	18			17			17		
CHO	14	18			17			17		
Nurse/Midwife	36	18			17			17		
**Tribe**										
Hausa	157	18	9.793	0.008*	17	5.045	0.080	17	5.103	0.028*
Fulani/Kanuri	33	16			15			16		
Others	30	20			18			18		
**Marital Status**										
Married	202	18	0.759	0.448	17	1.028	0.304	17	0.747	0.455
Single	18	19			18			18		
**Operation Hours**										
24 hours	82	18	9.954	0.005*	16	11.032	0.004*	17	5.535	0.043*
Daytime (8–6)	93	17			16			18		
Evening/ Morning only	45	19			18			17		
**Perceived Behavioural Control**										
Positive	186	20	4.020	0.001*	20	3.857	0.001*	20	3.903	0.001*
Negative	34	18			16			17		
**Subjective Norm**										
Present	208	18	0.418	0.676	17	0.331	0.741	17	0.339	0.734
Absent	12	16			16			16		
**Engaged in Other Business**										
None	42	19	2.042	0.7281	18			18		
Other jobs	38	18			17	5.532	0.237	17	4.629	0.328
Clinical	140	18			16			17		
**Age of Owner**	220	18	0.032	0.640	17	0.045	0.511	17	0.036	0.590
**Age of Shop**	220	18			17			17		
Under 6 years	113	18	1.661	0.097	17	0.645	0.519	17	0.656	0.512
6 years plus	107	17			16			17		
**Total**	220									

### Predictors of implementation measures

#### Acceptability

Table 3 presents the factors that predict the acceptability of intervention measure (AIM) among the respondents of the study. Findings revealed that individuals belonging to the tribes other than hausa, Fulani or kanuri showed significantly lower acceptability (p = 0.002) compared to the fulani/kanuri tribe (local estimate = 0.059, 95% CI [0.023, 0.081]). Also, Operating Hours influenced acceptability significantly (p = 0.001). Operating PPMV only in the evening or morning had a significantly lower effect on acceptability compared to those operating 24 hours (local estimate = 0.085, 95% CI [0.446, 0.903]).

**Table 3:** Predictors of acceptability of providing expanded BHS through PPMV.

Variable	Local Estimates (95% CI)	P-Value
**State of Residence**		
Jigawa (Reference)	1.000	0.097
Kaduna	0.250(0.011–0.428)	
**Tribe**		
Fulani/Kanuri (Reference)	1.000	-
Hausa	0.035(0.013–0.812)	0.113
Others	0.059(0.023–0.081)	0.002*
**Operating Hours**		
24 hours (Reference)	1.000	-
Daytime (8–6)	0.043(-0.673–1.324)	0.894
Evening/ Morning only	0.085(0.046–0.903)	0.001*
**Perceived Behavioral Control**		
Negative (Reference)	1.000	-
Positive	0.126(0.107–0.212)	0.010*

#### Self-reported reasons for accepting to provide expanded BHS

**Fig 2:**
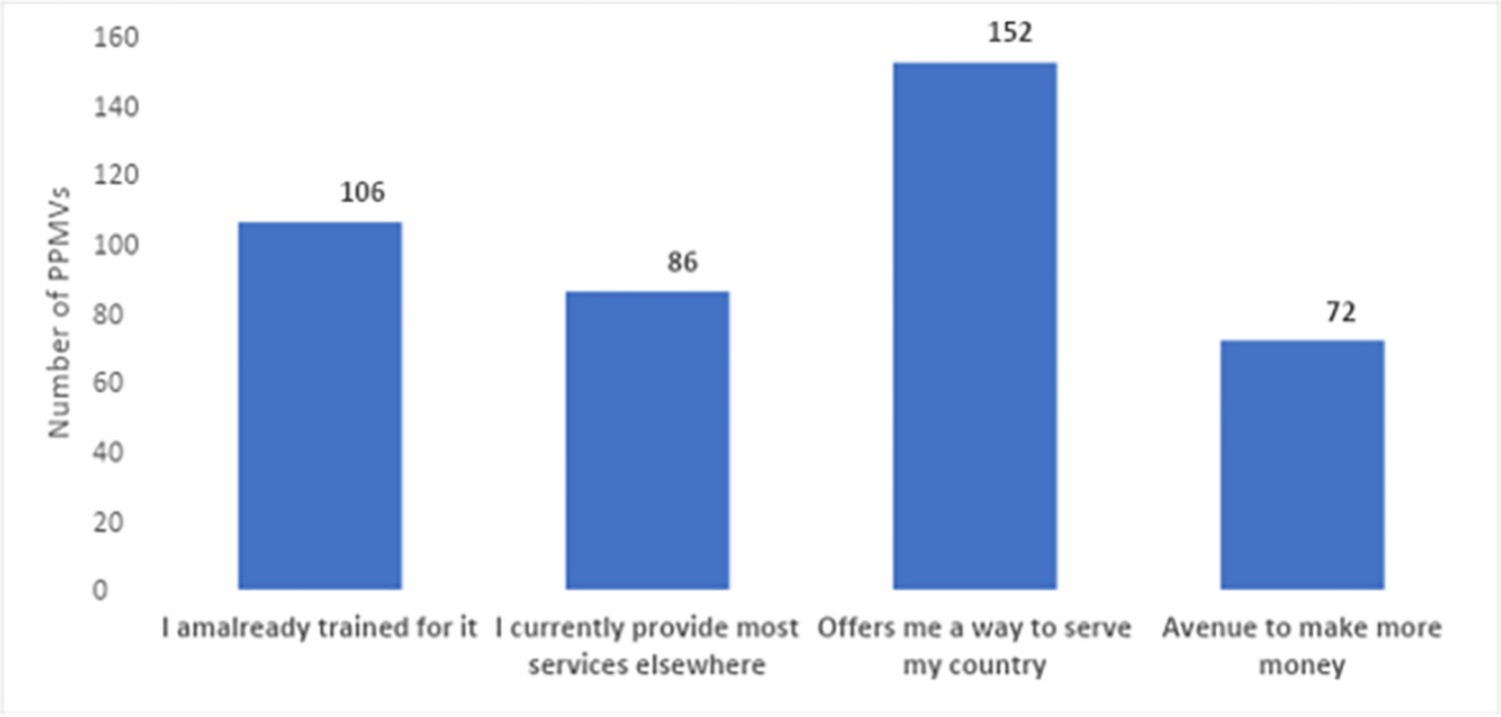
Self-reported reasons for acceptability (N = 198; Multiple responses).

We asked the 198 respondents who had maximum AIM score for the reasons behind their perception, their responses are summarized on Fig 2. Findings show that the desire to serve (152, 77%), and prior medical training (106, 54%) were the most popular reasons.

#### Appropriateness

The kernel regression analysis examined the factors influencing the appropriateness of provided expanded BHS, considering variables such as State of Residence, Sex, Operating Hours, and Perceived Behavioral Control (Table 4). State of Residence was found to be significantly associated with appropriateness (p = 0.001). Specifically, individuals residing in Kaduna showed a lower level of appropriateness compared to those in Jigawa (local estimate = 0.336, 95% CI [0.176, 0.551]). Operating Hours significantly influenced appropriateness (p = 0.001). PPMVs operating only during the evening or morning perceived providing expanded basic health services more appropriate compared to those operating 24 hours (local estimate = 1.318, 95% CI [1.045, 1.996]). Daytime operation (8–6) showed a non-significant influence on appropriateness compared to 24-hour services (local estimate = 0.596, 95% CI [0.195, 1.394], p = 0.133). Perceived Behavioral Control also significantly affected appropriateness (p = 0.001). Individuals with a negative perception of behavioral control exhibited lower appropriateness compared to those with a positive perception (estimated effect = 0.278, 95% CI [0.051, 0.321]).

**Table 4:** Predictors of appropriateness of providing expanded BHS through PPMV.

Variable	Local Estimates (95% CI)	P-Value
**State of Residence**		
Jigawa(Reference)	1.000	-
Kaduna	0.336(0.176–0.551)	0.001*
**Sex**		
Female(Reference)	1.000	-
Male	0.038(-0.006–0.022)	0.100
**Operating Hours**		
24 hours(Reference)	1.000	-
Daytime (8–6)	0.596(0.195–1.394)	0.133
Evening/ Morning only	1.318(1.045–1.996)	0.001*
**Perceived Behavioral Control**		
Positive (Reference)	1.000	-
negative	0.278(0.051–0.321)	0.001*

#### Feasibility

The kernel regression analysis identified significant factors influencing the feasibility, including Operating Hours and Perceived Behavioral Control (Table 5). Operating Hours significantly impacted the feasibility of intervention score (p = 0.030 for Daytime, p = 0.049 for Evening/Morning). Services operating exclusively during the daytime (8:00AM-6:00PM) or evening/morning were associated with lower feasibility of intervention measure (FIM) scores compared to those operating 24 hours Perceived Behavioral Control significantly affected feasibility of intervention measures (p = 0.002). Individuals with a negative perception of behavioral control tended to have lower levels of feasibility scores compared to those with a positive perception.

**Table 5:** Predictors of feasibility of providing expanded BHS through PPMV.

Variable	Local Estimates (95% CI)	P-Value
**State of Residence**		
Jigawa (Reference)	1.000	-
Kaduna	0.082(0.05–0.234)	0.350
**Tribe**		
Fulani/Kanuri (Reference)	1.000	-
Hausa	0.021(0.001–1.360)	0.484
Others	0.046(0.010–0.096)	0.085
**Operating Hours**		
24 hours (Reference)	1.000	-
Daytime (8–6)	0.734(0.028–0.960)	0.030*
Evening/ Morning only	0.706(0.103–0.931)	0.049*
**Perceived Behavioral Control**		
Positive (Reference)	1.000	-
Negative	0.067(0.020–0.611)	0.002*

## Discussion

This study reveals that expanding access to basic healthcare services in underserved communities through Patent and Proprietary Medicine Vendors (PPMVs) is not only highly acceptable but also feasible from the perspective of respondents. From our study of the 220 Medically trained PPMVs in underserved communities in the two Northern Nigerian states, we found that the PPMVs perceived the provision of expanded basic health services as more acceptable and feasible than appropriate. A mix of personal attributes of PPMVs and shop characteristics influenced the perception of PPMVs on the provision of expanded basic health services in the study population. We also reported that operating PPMV shop only in the evenings or morning hours significantly influenced all three implementation measures (i.e influences appropriateness positively; and acceptability and feasibility inversely) while perceived lack of behavioral control(a feeling of inadequate agency to decide on providing expanded basic health services) is related to the perceived acceptability, appropriateness and feasibility of providing expanded basic health services.

The remarkably high acceptability score aligns with findings from Ugbokwe et al. [33], where 97% of PPMVs expressed willingness to provide expanded basic healthcare services under a tiered accreditation initiative. Less than half of our respondents view their involvement in providing expanded basic healthcare services as appropriate, potentially due to the current limitation of their current service charter. Another possible explanation, suggested by Usar JI in 2020 [34], is that dissatisfaction among PPMVs with the existing regulatory framework, which is expected to be strengthened through the accreditation initiative, may have influenced their receptiveness.

Interestingly, PPMVs operating exclusively in the evening or morning demonstrated lower acceptability of providing expanded services. Fajola and colleagues [35] reported the high prevalence of dual employment as formal health worker and a PPMV. This could explain their hesitation about willingness to undertake the scrutiny associated with the provision of expanded basic health services. The dynamics in the acceptability of providing expanded basic health services by those primarily vending medicines and those who vend medicine as secondary occupation isn’t completely clear yet and therefore require further study in the future. An earlier study [33] alluded to the importance of profit making as a motivation (among other factors) for engaging in the business. This may have also been part of the factors contributing to the observed limited acceptability among PPMVs who do not operate full time.

Levesque and his co-authors [36] conceptualized access to healthcare through five domains: approachability, acceptability, availability/accommodation, affordability, and appropriateness. This framework has been widely employed to elucidate healthcare access by various authors [37] Previous studies have affirmed PPMVs’ approachability [38,39], availability/ accommodation [22], and affordability [40]. In further contributing to the existing body of knowledge on how PPMVs can contribute to improving healthcare access, our study reported high Acceptability (Median score 18 out of 20 obtainable), Appropriateness (Median score 17 out of 20 obtainable) as well as highlighting its feasibility (Median score 17 out of 20 obtainable).The statistically significant factors influencing implementation readiness for the PPMV tiering policy reported in our study closely align with the supply-side factors for improving healthcare access by influencing ability to seek, reach and engage healthcare services described by Levesque and Colleagues [36]. These include state of operation of PPMV (geographic location), tribe(socio-cultural), Operating hours (Hours of opening), perceived behavioral control(interpersonal), motivation to utilize professional skills to address community challenges (professional values).

Geographic location is one of the supply-side factors explained in the Levesque framework to determine ability of community residents to seek and reach healthcare services. We reported that state of practice **is** significantly associated with perceived appropriateness of providing expanded basic health services by the medically trained PPMVs. This finding underscores how differences in the conditions under which PPMVs operate in different states, such as the physical and business environment, and the support systems in place [36]). The state peculiarities, therefore, need to be adequately considered while defining framework for expanding access to basic health services through medically trained PPMVs to achieve its objectives.

Betancourt and colleagues [41] submitted that tribe reflects the cultural background and social norms of both the care providers and populations, and it can influence providers’ perceptions of the acceptability of healthcare services. Cu et al [37] posits that providers who close cultural ties with their patient populations or who have developed a strong understanding of these cultural contexts are more likely to feel comfortable delivering services that are culturally sensitive and acceptable, suggesting that fostering cultural competence and understanding among providers could improve service delivery outcomes. It is therefore important to fully consider tribal and other cultural peculiarities when designing frameworks or policies for expanding access to basic healthcare through medically trained PPMVs.

Operation hour was presented in the Levesque framework as a critical factor impacting the accessibility and accommodation of healthcare services [36]. PPMVs in our study differ in their perceived acceptability, appropriateness and feasibility of providing expanded basis healthcare services. It is known that limited or rigid operation hours can restrict patient access, especially for those who have inflexible schedules or face other time-related constraints [42,43]. To maximize the contribution of the medically trained PPMVs to improving healthcare access, the framework or policy should be appealing to PPMVs who have non or limited restriction in their time of operation.

Perceived behavioral control reflects healthcare providers’ confidence in their ability to independently decide to opt in for provision of expanded basic health services. These relate to the interpersonal factors that are associated with appropriateness in the Levesque framework [36]. When providers feel they have sufficient autonomy and control over their decision, they are more likely to participate in the eventual provision of the expanded healthcare services (National Institutes of Health, 2005). This factor, considered alongside the expressed enthusiasm- motivation to use professional skills to address the healthcare need of their community- presents a huge opportunity for substantial buy-in of the PPMVs because providers who are highly motivated and feel that their skills are being effectively utilized are more likely to embrace service expansion as an opportunity to apply their expertise in broader and more impactful ways. Leveraging this self-motivation and autonomy as an entry point for engagement of PPMVs and their associations during conception and rollout of service expansion initiative could lead enhance effective buy-in and rollout and substantially influence the success of the initiative.

In conclusion, based on evidence from the literature and our study regarding the five supply-side domains of healthcare access, there is significant potential for medically trained PPMVs operating in underserved communities in Northern Nigeria to contribute to bridging the gap in access to basic health services in hard-to-reach areas. For optimal success, the regulatory framework and accreditation system must be carefully designed in collaboration with PPMVs, the majority of whom expressed autonomy in decision to adopt service expansion initiatives and are motivated to utilize their skills to assist their communities. We recommend contextualizing the accreditation framework for expanding healthcare provision to medically trained PPMVs within each state, using an integrated hybrid bottom-up and top-down approach that combines grassroots and high-level perspectives to create more effective and inclusive policies [44–46].

This could include scoping sessions or workshops with local or state chapters of the National Association of Proprietary and Patent Medicine Dealers, medically trained PPMVs, residents of underserved communities, regulatory agencies and other relevant actors to receive inputs critical for maximum buy-in and success of the service expansion initiative. The output(s) of such scoping sessions may then be utilized to provide the national operational framework that each state can then contextualize to accommodate their peculiar socio-cultural factors, geographical landscape and the realities of their healthcare regulatory environment. The overall (National) design and processes for rollout should aim to preserve both the profitability of PPMVs as businesses and ensure the affordability of services for underserved communities.

## Limitation

Limitations of this study stem from its cross-sectional design, potentially reflecting only a snapshot of respondents’ experiences rather than their day-to-day realities. Furthermore, while the criteria for defining underserved communities were established in consultation with state-level stakeholders, caution is advised when comparing findings with studies utilizing different criteria. Additionally, the definition of "expanded basic health services" is framed within Nigeria’s PPMV tiering policy, implying that findings may not generalize to regions with dissimilar service expansion initiatives.

Differences in contextual definitions of target populations across varied settings may naturally lead to divergent outcomes. Despite these limitations, our study presents a unique perspective on a specific subgroup of PPMVs–those with medical training operating in medically underserved areas. This subgroup, central to many LMICs’ efforts to enhance healthcare access in underserved regions, has not been extensively explored in previous research.

The Authors couldn’t assess intra-rater reliability of the implementation measures used in this study (AIM, IAM and FIM) due to logistics difficulties related to availability of respondents and their dispersed distribution. However, the scales demonstrated good rating on the inter-rater reliability and internal consistency (Cronbach’s Alpha) which are consistent psychometric properties reported on the tools by previous authors

The insights provided by our study are poised to enhance ongoing endeavors to mitigate disparities in primary healthcare access within resource-constrained settings, particularly in Sub-Saharan Africa, where contexts akin to our study location are prevalent.

## Supporting information

S1 Checklist Inclusivity in global research. (DOCX)

S1 Data (DTA)
